# Récidive d'un pseudomyxome péritonéal avec métastases hépatique et splénique: à propos d'un cas rare avec revue de littérature

**DOI:** 10.11604/pamj.2018.30.225.15484

**Published:** 2018-07-25

**Authors:** Mustapha Azzakhmam, Fouad Zouaidia, Ahmed Jahid, Kaoutar Znati, Zakia Bernoussi, Najat Mahassini

**Affiliations:** 1 Laboratoire d’Anatomie Pathologique, Hôpital Militaire d’Instruction Mohamed V, Rabat, Faculté de Médecine et de Pharmacie de Rabat, Maroc; 2 Laboratoire d’Anatomie Pathologique, Centre Hospitalier Universitaire Avicenne, Rabat, Maroc

**Keywords:** Pseudo myxome péritonéal, métastase splénique, métastase hépatique, Peritoneal pseudo-myxoma, splenic metastasis, liver metastasis

## Abstract

Le pseudo myxome péritonéal (PMP), est un syndrome clinicopathologique caractérisé par une ascite mucineuse et des pools de mucine comportant un épithélium mucineux néoplasique dans la cavité péritonéale. Le PMP est peu fréquent, et se présente avec des manifestations cliniques et pathologiques inhabituelles posant des problèmes diagnostic et thérapeutiques. L'atteinte des viscères abdominaux et les métastases ganglionnaires sont rares et se limitent à des cas sporadiques rapportes dans la littérature. Nous rapportons ici le cas d'un patient de 56 ans opéré à deux reprises pour PMP d'origine appendiculaire, et qui a consulté pour douleurs abdominales évolutives, cinque ans après sa dernière cure. Le scanner avait objective une récidive de pseudo myxome péritonéal, avec présence de lésions intraparenchymateuses spléniques et hépatique .l 'étude anatomopathologique avait objective la récidive d'un pseudo myxome péritonéal de bas grade avec la localisation intrasplénique et hépatique des mêmes lésions histologiques confirmant les métastases.

## Abstract (en)

Pseudomyxoma peritonei (PMP) is a clinicopathologic syndrome characterized by mucinous ascites and pools of mucin resulting in neoplastic mucinous epithelium in the peritoneal cavity. PMP is uncommon and it is characterized by clinical and unusual pathologic manifestations posing diagnostic and therapeutic problems. Involvement of abdominal viscera and lymph node metastases are rare and sporadic cases have been reported in the literature. We here report the case of a 56-year old patient who had undergone two operations for PMP of appendicular origin presenting with progressive abdominal pain five years after his last treatment. Scanner objectified a recurrence of peritoneal pseudo-myxoma with liver and splenic intraparenchymatous lesions. Anatomopathological examination showed intrasplenic and hepatic recurrence of low-grade peritoneal pseudo-myxoma confirming the metastases.

## Introduction

Le pseudomyxome (PMP) péritonéal est un syndrome clinicopathologique qui se caractérise par une collection diffuse de fluide gélatineux dans la cavité péritonéale avec des implants gélatineux a la surface du péritoine. 94% des PMP se développent à partir de tumeurs mucineuses appendiculaires [1-4]. Cette entité pathologique est rare, son incidence rapportée dans la littérature est de l'ordre de 2/10.000 laparotomies avec prédominance masculine [1]. D'autres sites de départ du PMP ont été rapportés dans la littérature [5]. Même si le matériel gélatineux diffuse dans tout l'abdomen, l´invasion des organes de voisinage survient rarement, et la diffusion métastatique, aussi bien lymphatique que hématogène est extrêmement rare; de rares cas sporadiques sont rapportés dans la littérature et concernent des métastases aux vertèbres [6], aux poumons [7,8], au colon [9], aux ganglions lymphatiques [10,11] et à la rate [5,12]. Nous rapportons ici, un cas rare de PMP récidivant, avec métastases splénique et hépatique et dont le diagnostic a été oriente par la radiologie (scanner) et confirme par l'examen anatomo-pathologique des pièces opératoires.

## Patient et observation

**Figure 1:**
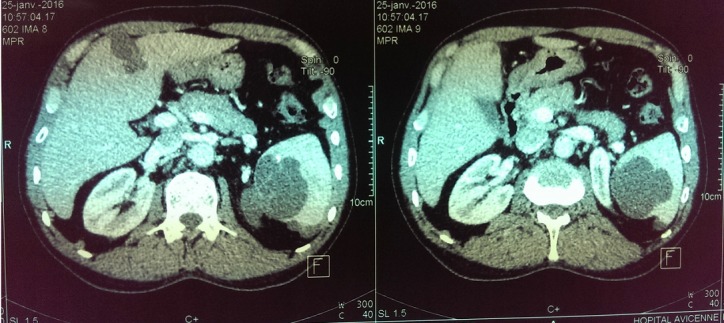
Scanner abdominal montrant des localisations spléniques et hépatiques du PMP, en absence d’enchâssement du foie et de la rate dans un matériel mucineux intra abdominal caractéristiques de métastases viscérales

**Figure 2:**
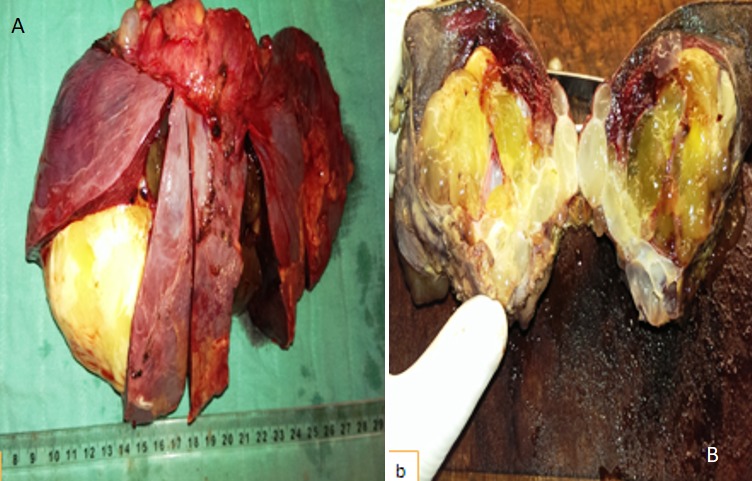
Aspect macroscopique de la rate: parenchyme splénique infiltré par une lésion gélatineuse prenant un aspect multiloculaire en surface

**Figure 3:**
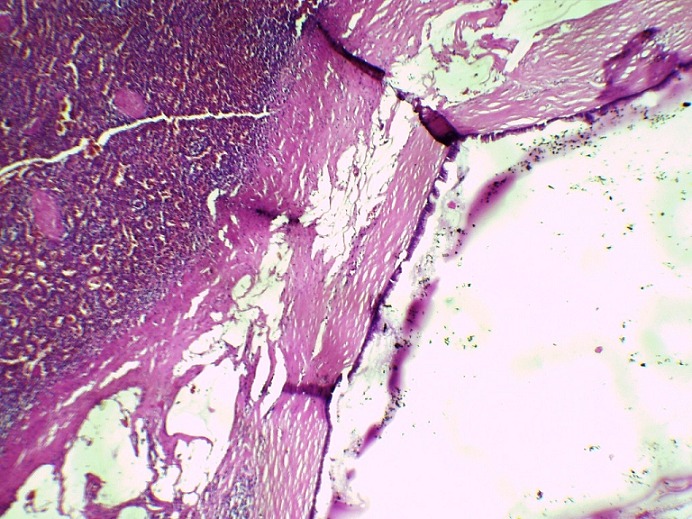
Flaques de mucus pauci-cellulaires bordées d ’un épithélium de bas grade cyto-nucléaire infiltrant le parenchyme splénique (HEx10)

**Figure 4:**
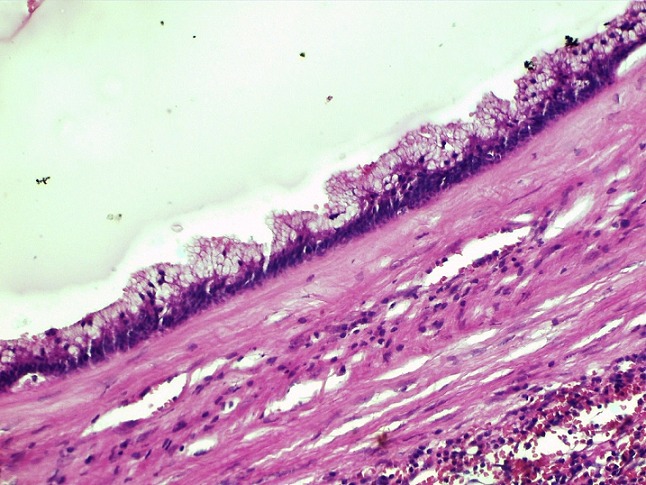
Épithélium muco-sécréteur montrant peu d’atypies cyto-nucléaires, et bordant des flaques de mucus pauci-cellulaires (HEx20)

**Figure 5:**
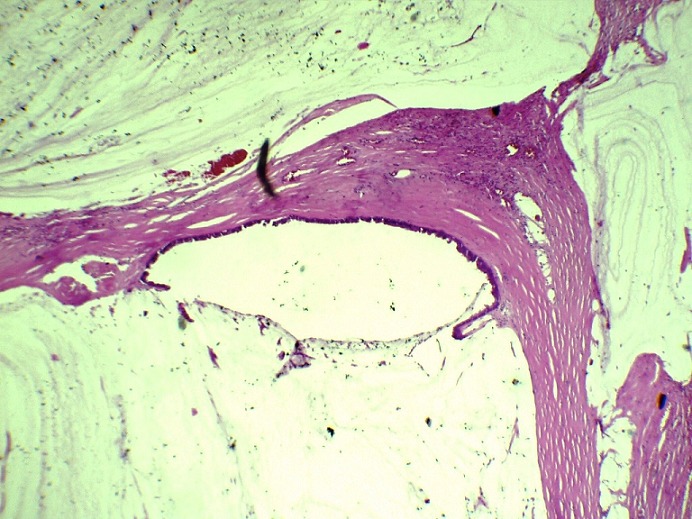
Flaques de mucus pauci-celllualire d’ un nodule péritonéal avec présence de lambeaux d’un épithélium de bas grade cyto-nucléaire témoignant de la récidive du PMP (HEX10)

**Figure 6:**
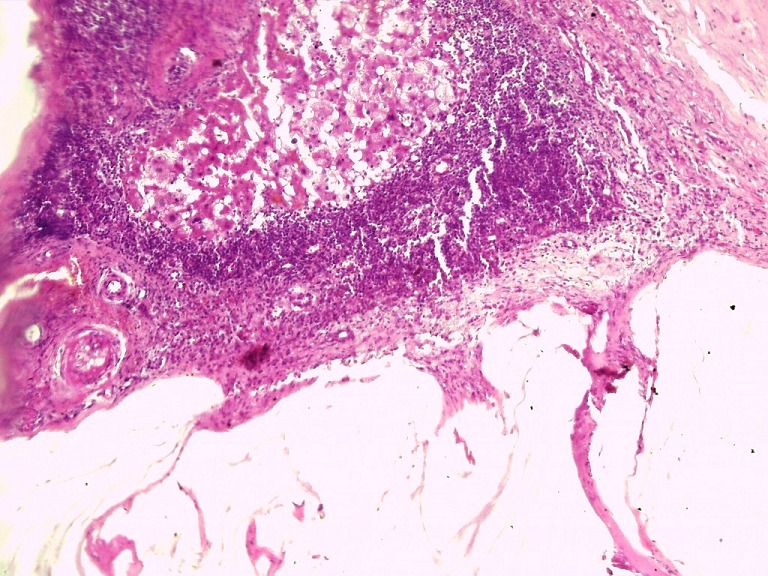
Flaques de mucine dissociant un parenchyme hépatique (HEx10)

Il s´agit d'un patient de 56 ans opéré déjà à deux reprises pour pseudomyxome péritonéal a point de départ appendiculaire, et qui a consulté dans notre formation cinque ans après sa dernière cure chirurgicale ,pour douleurs abdominales diffuses évoluant depuis plus de deux semaines en augmentant d´ intensité, sans irradiation et sans rythme particuliers, avec sensation de pesanteur au niveau de la région péri-ombilicale. L'examen clinique retrouva un patient conscient avec une tension artérielle a 12/07 et pouls a 80bpm. L'examen de l´abdomen avait montré une cicatrice de laparotomie médiane, la palpation notait une sensibilité au niveau de l´hypochondre droit et de la fosse iliaque droite, avec empâtement abdominal et splénomégalie moderee. Les orifices herniaires et les aires ganglionnaires étaient libres. Le reste de l'examen clinique était sans particularité. Devant ce tableau, plusieurs hypothèses diagnostic furent évoquées et notamment une récidive de son PMP. Le bilan biologique montrait des marqueurs tumoraux élevés avec un CA19-9 à 35ui/l et un ACE à 5ui/l. L´exploration radiologique par une échographie abdominale a montré un aspect de macro-kystes au niveau du ligament rond et de la rate. Le scanner abdominal avait confirmé la récidive au niveau péritonéale sous forme d'un nodule et présence de lésions similaire dans la rate en intra parenchymateux et au niveau du foie, sans enchâssement de ces organes par l ascite gélatineuse; aspect en faveur de métastases viscérales Figure 1. Le patient fut adresse en chirurgie ou il a subis une spleectomie avec exérèse des nodules péritonéaux et celui du ligament rond et les pièces anatomiques adressées au laboratoire d'anatomie pathologique. A l'étude anatomopathologique, l´envoi comportait trois pièces de résection chirurgicales, une pièce de splénectomie de 580grammes, mesurant 21x11x5cm. La rate était libre, a surface lisse laissant percevoir une capsule. Absence de lésions suspectes en surface et notamment de matériel gélatineux déposé en surface. A la coupe du parenchyme, issue d'une masse gélatineuse dense de grande abondance, cette lésion occupait toute l'épaisseur intra parenchymateuse sur 16x8x7cm, ne laissant persister en périphérie qu'un liseré de 5cm d'épaisseur, avec aspect multi kystique au contact de la capsule en surface sans la rompre Figure 2. Le deuxième fragment correspondait à un nodule accole à la paroi abdominale en regard du foie mesurant 1.5x1x0.5 cm. Le troisième fragment (nodule du ligament rond) mesurait 5x3x1.5cm, dont la coupe laissait une formation kystique gélatineuse bordée par un parenchyme hépatique. Histologiquement, de multiples prélèvements ont été effectués au niveau de la rate et ont montraient une prolifération faite de larges flaques de mucus pauci-cellulaires dissociant le parenchyme splénique en profondeur. Ces formations étaient bordées parfois d'un épithélium cylindrique simple, dont les cellules étaient dotées de noyaux en position basale et cytoplasme vacuolise, sans activité mitotique évidente et avec peu d'atypies cyto-nucléaires. Le parenchyme splenique adjacent montrait un aspect congestif Figure 3 et Figure 4. L'analyse du nodule accole à la paroi retrouvait également des flaques de mucus pauci-cellulaires Figure 5. Le nodule du ligament rond montrait un parenchyme hépatique siège des mêmes lésions histologiques décrites auparavant Figure 6. Ces aspects morphologiques ont permis de retenir le diagnostic d'une récidive d'un PMP de bas grade avec métastases spléniques et hépatiques.

## Discussion

La pathogénie du PMP a longtemps été débattue. Les premières théories concernaient la mucinification du méso-thélium abdominal, stimulée par la mucine elle-même [1-4]. Notre compréhension actuelle, est que la majorité des cas sont secondaires à des néoplasmes mucineux appendiculaires primitifs [1,3]. L´analyse moléculaire du K-RAS et la perte du caractère hétérozygote dans les tumeurs mucineuses appendiculaires et ovariennes synchrones, supportent l'origine appendiculaire de ces néoplasmes [13]. Dans les situations ou l´appendice est retrouve dilate ou rompue lors de la laparotomie, et que l'analyse histopathologique revele un carcinome dans l´appendice et dans l´ascite mucineuse, le diagnostic est simple [3]. Cependant, certains cas qui se présentent comme une masse pelvienne chez la femme, ou des patients qui n'ont pas une masse tumorale identifiable macroscopiquement, peuvent être un défi diagnostic. En 2010, l'American Joint Committee on Cancer (AJCC) et l'Organisation Mondial de Santé (WHO) , avaient subdivise le PMP en deux catégories en se basant sur le grade cytonucléaire de l'épithélium contenu dans la mucine: l'épithélium de bas garde définit l'adénocarcinome mucineux de bas grade et l´ épithélium de haut grade définit l'adénocarcinome mucineux de haut grade [1,3,13,14]. Cliniquement, le PMP peut se manifester par une distension abdominale, des douleurs abdominales secondaires à l´obstruction, ou des symptômes localises simulant une appendicite aigue. Chez les femmes, la symptomatologie peut être une pesanteur et ou des masses ovariennes palpables. Une hernie inguinale irréductible peut être un des symptômes chez les patients de sexe masculin [1]. le PMP est diagnostique incidentalement en per-opératoire, dans 20% des cas [15]. La plupart des patients auront des taux élevés de l´antigène carcino-embryonnaire(ACE) et du CA19-9, qui sont très utiles, aussi bien pour le diagnostic que pour le suivi de l´efficacité thérapeutique et la détection précoce des récidives survenant au cours du traitement [15]. Le CA 125 a été observé à des taux élevés chez les patientes avec atteinte ovarienne associée, son utilité est encore controversée [1]. En imagerie, l'échographie peut être trompeuse car l´ascite mucineuse pauci-cellulaire ressemble au fluide intra-péritonéal [2]. Le scanner par contre, est performant car les densités sont plus élevées par rapport aux ascites non mucineuses, et permet d'étudier l´extension en préopératoire [2]. Macroscopiquement, le PMP est reconnaissable lors de la laparotomie; il apparait sous forme de dépôt mucineux dans la cavité péritonéale. Dans de nombreux cas, il s'agit d´un constat inattendu au cours d´une laparotomie exploratrice [1,2]. Histologiquement, il se manifeste par la présence dans la cavité péritonéale de plages de mucine, avec des amas variables d´un épithélium néoplasique mucino-sécréteur; il peut s´agir d´une mucine pauci-cellulaire ou acellulaire, d’ une mucine organisée avec des capillaires et des fibroblastes proliférants, ou d'une mucine avec des cellules malignes flottantes dedans [1].

A l´étude immuno-hisochimique et moléculaire, les tumeurs d´origine appendiculaires expriment le Muc2 et le Muc5a, la CK20, le CDX2 et l'ACE et sont négatives pour la CK7 et le CA125. Ces immuno-marqueurs sont utiles pour déterminer le site de la tumeur primitive chez les patients avec un PMP [16]. L'augmentation de l´expression de la N-Cadherine et la diminution de l´expression de l'E-Cadherine, a été observée dans ces tumeurs. Ce décalage du phénotype cadherine, et l´expression de la vimentine peut traduire une transition épithélio-mésenchymateuse, transition lors de laquelle les cellules épithéliales acquièrent un phénotype mésenchymateux; processus promoteur de métastases [17]. D'autres marqueurs sont tres frequemment alteres dans les adénocarcinomes mucineux comme la Bêta-catenine, la Cycline D1, le Ki-67, le NF-kB, le VEGF, et la E-Cadherine et P53 [1]. Le rôle du pathologiste ne se limite pas à l´identification de mucine ou de l´épithélium muco- sécréteur; le premier objectif est d´identifier l´organe d'origine. Un néoplasme appendiculaire primitif étant le site de départ dans 94% des cas [1], ce qui correspond aussi à notre cas. Et bien que les métastases et ou l´invasion d´organes de voisinage soient très rares, le PMP manifeste habituellement des récidives à répétition et une progression insidieuse compromettant le fonctionnement du tractus digestif avec issue fatale. Les métastases à distance sont tres rares; la littérature contient uniquement des cas sporadiques de PMP avec métastases viscérales ou ganglionnaires; il s´agit de métastases aux vertèbres[16], poumon, colon [17], le tissu sous cutané de la paroi thoracique[18,19], les ganglions lymphatiques inguinaux [18], axillaires [19] et péri-aortiques [18] et enfin à la rate [5]. Un cas avait des métastases extensives au foie, au pancréas, a la vessie, aux ganglions péri-aortiques, à la plèvre et au péricarde [5]. En plus, l´invasion des organes de voisinage a été rapportée rare. Gough et al [19], avaient observé un PMP avec invasion secondaire d´organes chez 65% de leurs patients. C'est pour cette raison, qui ´il faut écarter l´éventualité d´une invasion secondaire directe due à de l'existence de collections de mucine néoplasique emprisonnant le viscère présumé site de métastase, avant de conclure à une métastase par voie lymphatique ou hématogène. En effet, Papavasiliou avait rapporté le cas d´invasion splénique par un PMP et avait noté la pénétration de la mucine collectée dehors, à travers le parenchyme splénique [20]. C'est dans cette situation, qu´apparait l'intérêt du scanner pré-opératoire et des constats lors de la laparotomie.

Dans le cas que nous rapportons, le scanner avait montré des lésions splénique et hépatique, en absence d'ascite mucineuse les englobant en périphérie. En plus, l´aspect histologique des lésions splénique et hépatique était identique et de même grade, que celui retrouve dans le nodule péritonéal. Dans une large série de 274 cas de PMP [3], cette atteinte de viscères abdominaux survenait aussi bien dans les lésions de haut grade, que celles de bas grade, il n´y avait aucune corrélation statistique entre le grade histologique de ces lésions et la fréquence d'invasion et de métastases viscérales et ganglionnaires; notre observation rejoint ces conclusions. Bien que les masses tumorales du PMP ne soient pas localement envahissantes, la mucine est localement destructrice et les complications résultent de la fibrose et de l´obstruction. Les lésions appendiculaires avec propagation au-delà de l´appendice sont associées à un PMP progressif, récidivant et fatal chez plus de 50% des patients [1]. Lorsqu´il est traité par des moyens principalement chirurgicaux, le pronostic du PMP est faible, avec une survie médiane de 2 ans et une survie à 5 ans de 53% à 75% [1-3] . La péritonéotomie et la chimiothérapie intrapéritonéale ont augmenté la survie à 10 ans à la fin des années 1990, chez 80% des patients dans une grande série [1], et la cytoréduction avec chimiothérapie intrapéritonéale hyperthermique (HIPEC) a démontré une amélioration de la maladie à 5 ans de survie chez 56% à 75% des patients. De meilleurs taux sont atteints dans les centres spécialisés. La thérapie agressive a un risque de mortalité postopératoire allant jusqu´à 5%, et un taux de morbidité sévère allant jusqu´à 40% [1-3, 10, 19,20]. La gestion agressive entraîne la plus grande amélioration de la survie chez les patients avec un diagnostic de maladie de bas grade. La chimiothérapie systémique à une efficacité limitée sur le PMP, et elle est généralement réservée aux patients atteints d´une maladie progressive ou récidivante [3]. La persistance du PMP malgré une thérapie locale agressive, associée à la morbidité et à la mortalité liée à la chirurgie de cytoréduction avec HIPEC, a suscité l´intérêt des thérapies ciblées, en particulier celles visant la production de mucine [1,3]. Vu les récidives chez notre patient, et la survenu de métastases splénique et hépatique, une chimiothérapie systémique a été décidée conformément a aux recommandations; le patient a été mis sous 5 Fluouracile, Folinates de calcium, Irinotecan (protocole FOLFIRI). Aux premières réévaluation ,le patient se portait bien et ne montrait pas de signes de récidives de la maladie sur le contrôle par scanner abdominal. Il est actuellement suivi au Centre National d'Oncologie de Rabat.

## Conclusion

En conclusion, le PMP péritonéal est une lésion néoplasique rare et dont la pathogénie n'est pas totalement élucidée. Les invasions d'organes et des métastases viscérales sont rares et se limitent à des cas sporadiques rapportes dans la littérature. Notre cas rejoint ces observations rapportées.

## Conflits d’intérêts

Les auteurs ne déclarent aucun conflit d'intérêts.
